# Avelumab Maintenance Therapy for Advanced Urothelial Carcinoma with Low Tumor Burden

**DOI:** 10.3390/cancers17152447

**Published:** 2025-07-23

**Authors:** Nobuki Furubayashi, Jiro Tsujita, Azusa Takayama, Shin Nakashima, Motonobu Nakamura, Takahito Negishi

**Affiliations:** Department of Urology, NHO Kyushu Cancer Center, Fukuoka 811-1395, Japann2takajin@gmail.com (T.N.)

**Keywords:** urothelial carcinoma, avelumab, maintenance therapy, low tumor burden, lymph node metastasis, visceral metastasis, platinum-based chemotherapy, prognostic factor

## Abstract

**Background/Objectives:** Commonly characterized by limited metastatic sites, low tumor burden has been associated with favorable patient outcomes in various malignancies. However, its prognostic relevance in avelumab maintenance therapy for advanced urothelial carcinoma (UC) remains incompletely defined. **Methods:** We retrospectively analyzed 26 patients with advanced UC who received avelumab maintenance therapy following disease control with first-line platinum-based chemotherapy between March 2021 and May 2025. Patients were categorized by their metastatic pattern at a chemotherapy initiation: lymph node-only (as a surrogate for low tumor burden), non-visceral (excluding visceral organ involvement, but including bone), or visceral disease. Survival outcomes were assessed using the Kaplan–Meier method. **Results:** Among the cohort, 46.2% had lymph node-only metastasis and 57.7% had non-visceral disease. The median progression-free survival (PFS) and overall survival (OS) from the start of avelumab were 5.6 months and 21.7 months, respectively. OS from the initiation of platinum-based chemotherapy was 28.7 months. Patients with lymph node-only metastasis demonstrated significantly longer OS from chemotherapy initiation compared with those with other metastatic patterns (41.1 vs. 22.9 months, *p* = 0.044). However, the PFS and OS from avelumab initiation did not significantly differ. No survival benefit was observed for patients with non-visceral disease compared with those with visceral metastases. **Conclusions:** Lymph node-only metastasis, representing low tumor burden, was associated with significantly improved long-term survival in advanced UC patients undergoing avelumab maintenance following chemotherapy. These findings support the clinical utility of baseline tumor burden and metastatic pattern in risk stratification and shared decision-making for maintenance therapy in advanced UC.

## Simple Summary

Avelumab maintenance therapy is a standard treatment for advanced urothelial carcinoma (UC) after an initial response to platinum-based chemotherapy. However, it remains unclear which patient subgroup benefits most. This retrospective study investigated the role of metastatic pattern, used as a surrogate for tumor burden, in predicting survival. Among 26 advanced UC patients treated with avelumab between 2021 and 2025, nearly half had lymph node-only metastasis, while others had either non-visceral or visceral disease. Patients with lymph node-only metastasis showed significantly longer overall survival from the start of chemotherapy (41.1 vs. 22.9 months), though this benefit was not observed when measured from avelumab initiation. Importantly, those with non-visceral disease, including bone involvement, did not show improved outcomes compared with those with visceral metastasis. These results suggest that lymph node-only metastasis may represent a favorable prognostic subgroup and could aid in guiding treatment discussions and personalized decisions for advanced UC maintenance therapy.

## 1. Introduction

The therapeutic landscape for advanced urothelial carcinoma (UC), encompassing both locally advanced and metastatic forms, has undergone major changes in recent years, leading to a broad array of treatment options. According to guidelines from the National Comprehensive Cancer Network, several combination regimens are currently recommended as category 1 options for systemic therapy in advanced UC cases [1]. Enfortumab vedotin (EV) in combination with pembrolizumab is listed as the preferred first-line regimen [2]. Additionally, the triplet regimen of gemcitabine, cisplatin, and nivolumab followed by nivolumab maintenance is recommended as an alternative [3]. Furthermore, the previous approach, gemcitabine and cisplatin followed by avelumab maintenance, continues to be endorsed as a category 1 regimen [4]. Although all three regimens are supported by the highest level of clinical evidence, the optimal selection of patients for each treatment strategy remains unclear. These regimens differ in both efficacy and safety profiles, necessitating individualized treatment decisions based on various clinical factors such as performance status, renal function, comorbidities, and patient preferences. Accordingly, determining the most appropriate regimen in real-world clinical practice remains a significant challenge [5].

Avelumab is a fully human IgG1 monoclonal antibody that targets programmed death-ligand 1 (PD-L1), thereby restoring antitumor immune responses by inhibiting the PD-1/PD-L1 immune checkpoint pathway [6]. In the phase III JAVELIN Bladder 100 trial, avelumab maintenance therapy significantly prolonged overall survival compared with best supportive care in patients whose disease had not progressed after first-line platinum-based chemotherapy, establishing its role as a standard maintenance treatment in this setting [4]. Given its manageable safety profile and clinical benefits, avelumab is particularly suitable for long-term use in patients with preserved performance status following chemotherapy. Subsequent real-world studies have confirmed the feasibility of avelumab therapy and highlighted durable responses in selected patient subsets. In particular, evidence suggests that patients with low tumor burden, such as those with lymph node-only or non-visceral metastases, may experience prolonged benefit from maintenance immunotherapy [7,8].

While these developments have expanded the treatment choices for UC, the clinical significance of metastatic distribution at the time of treatment initiation, such as lymph node-only or non-visceral disease, remains incompletely defined. Notably, avelumab has been established as a maintenance immunotherapy initiated after first-line platinum-based chemotherapy in patients without evidence of disease progression, yet its efficacy in distinct metastatic patient subgroups has not been fully characterized in real-world practice.

Through a retrospective observational analysis, we investigated the outcomes of avelumab maintenance therapy in patients diagnosed with advanced UC, with a particular focus on the prognostic implications of metastatic distribution at baseline. To support more individualized treatment strategies under shared decision-making, we aimed to clarify whether the presence of lymph node-only or non-visceral metastases could influence survival outcomes in these patients.

## 2. Patients and Methods

### 2.1. Patient Population

We retrospectively identified 26 consecutive patients with advanced UC (either locally advanced or metastatic) who received avelumab as maintenance therapy following first-line platinum-based chemotherapy without evidence of disease progression between March 2021 and May 2025 at Kyushu Cancer Center, Japan.

Avelumab was administered intravenously at a dosage of 10 mg/kg every two weeks, which was continued until radiologic or clinical disease progression or the development of unacceptable toxicity, in accordance with previously established protocols [4,9]. No study-specific procedures or additional clinical assessments were mandated. Patients were monitored as per routine clinical practice, with follow-up schedules determined at the discretion of the treating physician [10]. The best response to platinum-based chemotherapy and avelumab treatment was evaluated based on tumor response as defined by the Response Evaluation Criteria in Solid Tumors, version 1.1 [11]. Relevant clinical information was obtained from electronic medical records for each patient.

In this study, lymph node-only metastasis was defined as the involvement of lymph nodes, irrespective of the presence or absence of residual primary tumors in the urinary tract. Non-visceral metastasis was defined as the absence of metastases to visceral organs, not considering bone metastasis. This definition was applied because the cohort excluding patients with lymph node-only metastasis overlapped entirely with the group categorized as having visceral metastases when bone lesions were considered part of visceral spread.

This study was conducted with approval from the Ethics Review Committee of the National Hospital Organization Kyushu Cancer Center (Approval No. 2014-99) and complied with the principles outlined in the Declaration of Helsinki. Because of the retrospective design of this study, informed consent was obtained through an opt-out approach.

### 2.2. Statistical Methods and Analytical Approach

All statistical procedures were conducted using EZR (version 1.68; Saitama Medical Center, Jichi Medical University, Saitama, Japan) [12]. Progression-free survival (PFS) was defined as the time from the initiation of avelumab maintenance therapy to the date of investigator-assessed clinical and/or radiographic disease progression or death from any cause, whichever occurred first. Overall survival (OS) was defined as the time from the initiation of avelumab maintenance therapy to death from any cause. For OS calculated from the initiation of platinum-based chemotherapy, the same criteria were applied, with the start date adjusted accordingly. Survival outcomes were estimated using the Kaplan–Meier method. Patients who had not experienced an event by the data cutoff date were censored at the time of their last follow-up. Differences in survival between subgroups were compared using the log-rank test. A two-sided *p*-value of less than 0.05 was considered statistically significant.

## 3. Results

### 3.1. Patient Characteristics

The clinical baseline features observed in 26 patients diagnosed with advanced UC at the commencement of first-line platinum-based chemotherapy are summarized in Table 1. All patients included in this analysis showed no evidence of disease progression following chemotherapy and subsequently received avelumab maintenance therapy, in accordance with the eligibility criteria of the JAVELIN Bladder 100 trial.

The median age was 72 years (interquartile range [IQR], 65–77), with 17 patients (65.4%) being male and 9 (34.6%) females. The Eastern Cooperative Oncology Group (ECOG) performance status was 0 in 22 patients (84.6%), and ≥1 in 4 patients (15.4%). The primary tumor site was located in the lower urinary tract in 14 patients (53.8%), the upper urinary tract in 10 (38.5%), and both regions in 2 (7.7%). Surgical resection of the primary tumor had been performed in 13 patients (50.0%). Histopathological analysis revealed pure UC in 19 patients (73.1%), while the remaining 7 (26.9%) exhibited subtype histologic features. Regarding metastatic distribution, the most frequently involved site was lymph nodes (n = 21, 80.8%), followed by the primary tumor organ (pelvis, ureter, or bladder; n = 13, 50.0%), lung and bone (each n = 4, 15.4%), peritoneal dissemination (n = 3, 11.5%), liver, brain, and other sites such as uterus or heart (each n = 2, 7.7%). Based on our study-specific definitions, 12 patients (46.2%) had lymph node-only metastasis, and 15 patients (57.7%) were classified as having non-visceral metastasis.

With respect to treatment response, 15 patients (57.7%) achieved either a complete response or partial response to platinum-based chemotherapy, while 11 patients (42.3%) exhibited stable disease. No patients experienced disease progression prior to the initiation of avelumab maintenance therapy.

### 3.2. Clinical Outcomes in the Overall Study Population

The median follow-up duration from the initiation of platinum-based chemotherapy was 16.2 months (IQR, 7.4–24.9). At the time of analysis, 5 patients (19.2%) remained on avelumab maintenance therapy, while 14 patients (53.8%) had died during follow-up.

**Figure 1:**
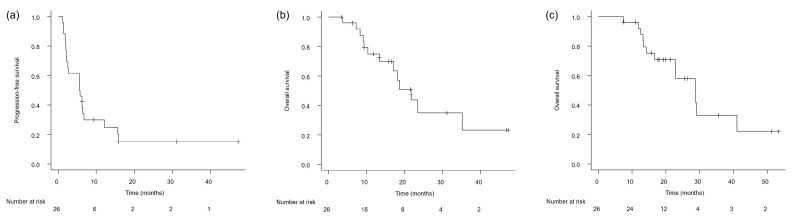
Kaplan–Meier survival curves in the overall study population with advanced urothelial carcinoma: (**a**) Progression-free survival from the initiation of avelumab maintenance therapy. (**b**) Overall survival from the initiation of avelumab maintenance therapy. (**c**) Overall survival from the initiation of first-line platinum-based chemotherapy.

The median PFS and OS from the start of avelumab maintenance were 5.6 months (95% confidence interval [CI], 2.2–6.8) and 21.7 months (95% CI, 13.3–35.1), respectively (Figure 1a,b). Additionally, the median OS, when calculated from the initiation of first-line platinum-based chemotherapy, was observed to be 28.7 months (95% CI, 16.6–41.1) (Figure 1c).

### 3.3. Clinical Outcomes in Patients with Lymph Node-Only and Non-Visceral Metastases

**Figure 2:**
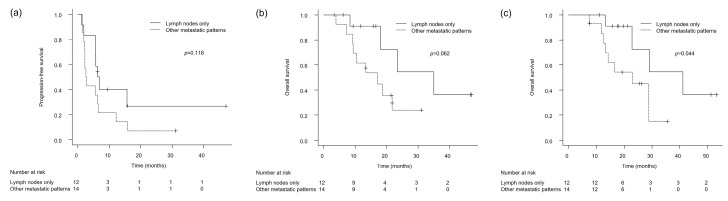
Kaplan–Meier survival curves stratified by metastatic pattern (lymph node-only vs. other metastatic sites): (**a**) Progression-free survival (PFS) from the initiation of avelumab maintenance therapy. (**b**) Overall survival (OS) from the initiation of avelumab maintenance therapy. (**c**) Overall survival (OS) from the initiation of first-line platinum-based chemotherapy. *p*-values were calculated using the log-rank test.

The log-rank test revealed no statistically significant difference in PFS following the initiation of avelumab maintenance treatment between patients with lymph node-only metastasis and those with other metastatic sites (6.5 vs. 2.6 months, *p* = 0.118) (Figure 2a). Similarly, OS following the start of avelumab maintenance did not show a statistically meaningful difference between the two groups (35.1 vs. 17.0 months, *p* = 0.062) (Figure 2b). However, when OS was assessed from the initiation of first-line platinum-based chemotherapy, patients with lymph node-only metastasis experienced significantly longer survival compared with those with other metastatic patterns (41.1 vs. 22.9 months, *p* = 0.044) (Figure 2c).

**Figure 3:**
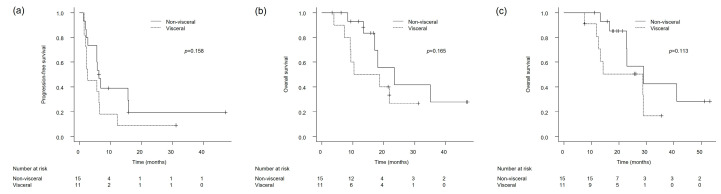
Kaplan–Meier survival curves stratified by metastatic burden (non-visceral vs. visceral metastases): (**a**) Progression-free survival (PFS) from the initiation of avelumab maintenance therapy. (**b**) Overall survival (OS) from the initiation of avelumab maintenance therapy. (**c**) Overall survival (OS) from the initiation of first-line platinum-based chemotherapy. *p*-values were calculated using the log-rank test.

The log-rank test indicated that PFS, measured from the start of avelumab maintenance therapy, did not differ significantly between patients with non-visceral and visceral metastases (6.2 vs. 2.6 months, *p* = 0.158) (Figure 3a). Likewise, OS calculated from the commencement of avelumab treatment did not differ meaningfully between these groups (23.4 vs. 14.4 months, *p* = 0.165) (Figure 3b). When OS was evaluated from the commencement of initial platinum-based chemotherapy, no statistically significant difference was identified between patients presenting with non-visceral metastases and those with visceral involvement (29.0 vs. 28.7 months, *p* = 0.113) (Figure 3c).

## 4. Discussion

This retrospective observational analysis explored the clinical efficacy of avelumab as a maintenance therapy in patients with advanced UC, with a particular emphasis on those who presented with lymph node-only or non-visceral metastatic involvement at the initiation of first-line platinum-based chemotherapy. This subgroup was selected because avelumab is used as switch maintenance therapy in patients without disease progression after first-line treatment, and both patients and physicians often consider metastatic status when choosing a treatment strategy before initiating systemic therapy [13]. Our results suggested that patients with lymph node-only metastasis, regardless of the presence of residual primary tumors, appeared to have longer OS when calculated from the start of platinum-based chemotherapy relative to those with alternative metastatic distributions. However, when measured from the initiation of avelumab maintenance, both PFS and OS did not show a statistically significant difference between the two groups. Additionally, patients with non-visceral metastases, including bone involvement, did not show a clear survival advantage over those with visceral disease. These findings were consistent across all endpoints evaluated: PFS and OS from the start of avelumab and OS from the start of first-line chemotherapy.

The era of shared decision-making has become increasingly feasible, even for patients with advanced UC, as the expansion of therapeutic options now empowers patients to actively participate in treatment selection. Under this model, clinical decisions should incorporate not only efficacy and safety data, but also an individualized assessment of the patient’s circumstances, such as lifestyle, occupational responsibilities, treatment schedule preferences, financial burden, and personal goals of care [14]. Importantly, the ultimate therapeutic goal is to achieve meaningful clinical benefit while minimizing treatment-related toxicity. In certain cases, it may be preferable to choose a regimen with a more favorable safety profile, even if it does not demonstrate the highest objective response rate reported in the trials. If comparable survival outcomes can be achieved with fewer adverse events, such strategies may benefit both patients and physicians responsible for managing treatment toxicity. This patient-centered perspective emphasizes the importance of balancing efficacy and quality of life, particularly in the long-term management of advanced malignancies.

The concept of low tumor burden is increasingly recognized as a clinically relevant prognostic and predictive factor across urologic malignancies. However, its definition and significance vary depending on the tumor type, biological behavior, and treatment context, even within the urologic oncology field [4,15,16,17]. In advanced UC, low tumor burden is most commonly defined qualitatively, including with lymph node-only metastasis, non-visceral metastasis, or a limited number of metastatic sites. These features are generally considered surrogate markers of a less aggressive disease phenotype and are associated with improved outcomes [4,15,18,19,20].

In the evolving treatment landscape of advanced UC, two pivotal trials, JAVELIN Bladder 100 and EV-302, have demonstrated the efficacy of novel regimens in distinct clinical settings. In the pivotal phase III JAVELIN Bladder 100 study, patients who received avelumab as maintenance therapy following completion of first-line platinum-based chemotherapy achieved a median overall survival of 23.8 months [21]. Notably, patients with lymph node-only metastasis demonstrated particularly favorable outcomes, achieving a median OS of 31.9 months and a median PFS of 8.7 months [22]. The regimen was well tolerated, with grade ≥ 3 treatment-related adverse events (TRAEs) observed in 47.4% and treatment discontinuation in only 11.9% of patients [4]. In contrast, the EV-302 trial evaluated EV plus pembrolizumab as first-line therapy, reporting a median OS of 33.8 months in the overall cohort. The outcomes were particularly favorable among patients with lymph node-only metastasis, with a median PFS of 22.1 months and median OS not reached at the data cutoff (median follow-up: 29.1 months) [23]. However, toxicity was more pronounced in this trial, with grade ≥3 TRAEs in 67.5% and treatment discontinuation in 35.1% of patients [2].

Our retrospective analysis supports these findings. Among the patients treated with avelumab maintenance, those with lymph node-only metastasis achieved a median OS of 41.1 months from the start of platinum-based chemotherapy. While PFS and OS from the initiation of avelumab were not significantly different compared with those with other metastases, the favorable OS trend in the lymph node-only group may indicate durable disease control with a less intensive regimen. Taken together, these results reinforce lymph node-only metastasis as a favorable-risk characteristic, associated with excellent long-term outcomes. While EV plus pembrolizumab offers substantial clinical benefit, the higher toxicity profile may limit its use in certain populations. For appropriately selected patients, such as those with low tumor burden, good performance status, and lymph node-only disease, avelumab maintenance remains a compelling and less toxic alternative, especially within the framework of shared decision-making.

The definition of “visceral” remains ambiguous in clinical oncology. According to the National Cancer Institute’s Dictionary of Cancer Terms, “visceral” refers to the viscera, the soft internal organs of the body, including the lungs, heart, and organs of the digestive, excretory, reproductive, and circulatory systems [24]. However, in the context of metastatic UC, the classification of metastatic sites as “visceral” is not standardized across clinical trials or retrospective studies. This inconsistency complicates the interpretation of subgroup analyses and application of clinical trial data to real-world settings. In the JAVELIN Bladder 100 trial, the patients were stratified by metastatic site at the initiation of first-line platinum-based chemotherapy, distinguishing between visceral and non-visceral disease. In that trial, non-visceral disease included locally advanced tumors, lymph node-only metastases, and bone metastases [22]. In contrast, the EV-302 trial, which evaluated EV plus pembrolizumab in previously untreated advanced UC cases, classified bone metastases as visceral involvement [2]. These differing definitions have important implications for how efficacy outcomes are interpreted across studies, as well as how treatment strategies are formulated in clinical practice. In our study, we adopted a definition consistent with that used in the JAVELIN Bladder 100 trial, classifying bone metastases as non-visceral. This approach was chosen because when the patients with lymph node-only metastasis were excluded, this group completely overlapped with those categorized as having visceral metastases when bone lesions were considered visceral, which would have confounded subgroup distinction. This approach allowed for a more direct comparison with prior avelumab-based data and enabled a clearer analysis of the prognostic relevance of lymph node-only disease. However, the variation in definitions across studies underscores the urgent need for a consensus in classifying UC metastatic patterns, especially as treatment paradigms continue to evolve.

Visceral metastasis has long been recognized as an indicator of poor prognosis in multiple malignancies. However, the term “visceral” is inconsistently applied, sometimes restricted to parenchymal organs, such as the liver or lung, and at other times broadened to encompass bone because of its uniformly adverse prognostic impact. Indeed, bone metastases are consistently associated with inferior survival in a range of solid tumors, including breast, lung, and prostate cancers, as well as renal cell carcinoma and UC [25,26,27,28]. In UC, specifically, bone involvement has been shown to be an independent predictor of shorter OS when compared with other metastatic sites [28,29,30]. Accordingly, when visceral disease is employed as a surrogate for high-risk biology, it is reasonable, particularly in UC, to classify bone metastasis as visceral on the basis of its established prognostic significance. Alternatively, a simpler and clinically intuitive approach, dichotomizing patients into “lymph-node-only” and “all other metastases” (encompassing both bone and visceral sites) groups, may yield sharper prognostic discrimination and therefore better guide therapeutic decision-making.

This study has several limitations that should be acknowledged. First, its retrospective design and single-center setting inherently introduce potential biases in patient selection, clinical management, and data interpretation. Second, the small sample size limits the statistical power to detect subtle differences between subgroups, particularly in analyses comparing non-visceral and visceral metastases. Third, our classification of metastatic sites, particularly the designation of bone metastases as non-visceral, was based on definitions used in prior studies, such as the JAVELIN Bladder 100 study. However, the lack of a universally accepted definition of “visceral metastasis” complicates comparisons across studies and may limit the generalizability of our findings. Additionally, all patients were recruited from a single institution in Japan and were ethnically homogeneous, which may limit the applicability of our results to more diverse populations. Genomic profiling data were also unavailable for most patients, precluding analyses of molecular subtypes or predictive biomarkers that could further inform treatment outcomes.

Despite these limitations, our results offer meaningful real-world insights into the prognostic relevance of metastatic distribution, especially the favorable outcomes associated with lymph node-only disease, in UC patients receiving avelumab maintenance therapy.

## 5. Conclusions

This study suggests that UC patients with lymph node-only metastasis appeared to be associated with improved long-term survival when treated with avelumab maintenance following platinum-based chemotherapy. While PFS and OS during avelumab therapy did not significantly differ, OS from first-line treatment was notably longer in the lymph node-only metastasis subgroup. These findings support the value of metastatic distribution, especially lymph node-only disease, as a prognostic factor in guiding maintenance treatment strategies for UC.

## Figures and Tables

**Table 1:** Baseline characteristics of patients with advanced urothelial carcinoma treated with avelumab maintenance, assessed at the initiation of platinum-based chemotherapy.

Characteristic (n = 26)	
Age (years), median (IQR)	72 (65–77)
Sex, no. (%)	
Male	17 (65.4)
Female	9 (35.6)
ECOG PS score, no. (%)	
0	22 (84.6)
≥1	4 (15.4)
Primary tumor site, no. (%)	
Upper urinary tract	10 (38.5)
Lower urinary tract	14 (53.8)
Both	2 (7.7)
Surgical treatment for the primary tumor, no. (%)	13 (50.0)
Pure UC in histologic testing, no. (%)	19 (73.1)
Disease site, no. (%)	
Lymph node	21 (80.8)
Primary tumor organ (pelvis, ureter, and bladder)	13 (50.0)
Lung	4 (15.4)
Bone	4 (15.4)
Peritoneal dissemination	3 (11.5)
Liver	2 (7.7)
Brain	2 (7.7)
Others (uterus or heart)	2 (7.7)
Response for platinum-based chemotherapy, no. (%)	
CR + PR	15 (57.7)
SD	11 (42.3)

IQR, interquartile range; ECOG PS, Eastern Cooperative Oncology Group performance status; UC, urothelial carcinoma; CR, complete response; PR, partial response; SD, stable disease.

## Data Availability

The data that support the findings of this study are available from the corresponding author upon reasonable request.
